# Parsimonious Models for Predicting Mortality from Choroidal Melanoma

**DOI:** 10.1167/iovs.61.4.35

**Published:** 2020-04-25

**Authors:** Bertil Damato, Antonio Eleuteri, Rumana Hussain, Helen Kalirai, Sophie Thornton, Azzam Taktak, Heinrich Heimann, Sarah E. Coupland

**Affiliations:** 1 Nuffield Laboratory of Ophthalmology, Nuffield Department of Clinical Neuroscience, University of Oxford, Oxford, United Kingdom; 2 Ocular Oncology Service, Royal Liverpool University Hospital, Liverpool, United Kingdom; 3 Liverpool Ocular Oncology Research Group, Institutes of Translational Medicine, University of Liverpool, Liverpool, United Kingdom; 4 Department of Medical Physics and Clinical Engineering, Liverpool University Hospital, Liverpool, United Kingdom; 5 Liverpool Clinical Laboratories, Royal Liverpool University Hospital, Liverpool, United Kingdom

**Keywords:** uveal melanoma, mortality, prognostication

## Abstract

**Purpose:**

To develop parsimonious models for estimating metastasis mortality in patients with choroidal melanoma for situations where use of the Liverpool Uveal Melanoma Prognosticator Online (LUMPO) or Tumor, Node, Metastasis (TNM) staging system is not possible.

**Methods:**

A backward-selection algorithm identified largest basal tumor diameter (LBTD) and chromosome 3 status (C3S) as the most informative predictors of metastatic death. We defined two prognostic models, based on LBTD with or without known C3S, that took into account competing risks of death from other causes by using the Aalen estimator. The bootstrap procedure was used to estimate discrimination accuracy, expressed by the C-index.

**Results:**

The cohort was comprised of 8348 patients with choroidal melanoma, 4174 of whom had known chromosome 3 status; of the 1553 deaths that occurred among these patients, 956 were attributed to metastasis. For LBTD with or without known C3S, the metastatic-death-specific C-indices at 2, 5, and 10 years were 0.85, 0.85, and 0.84 and 0.79, 0.77, and 0.74, respectively, as compared with 0.81, 0.79, and 0.76 for Kaplan–Meier prognostication using the 8th edition of the TNM staging system.

**Conclusions:**

We have developed parsimonious models for predicting the absolute risks of metastatic death from choroidal melanoma that take into account competing causes of death and which compare favorably with the current version of the TNM staging system. There is a need for further studies to validate the use of these models in situations where use of the TNM or LUMPO is not possible.

Almost 50% of patients with choroidal melanoma develop metastatic disease, which is almost always fatal.^1^ If sufficiently reliable, mortality prognostication can provide reassurance to patients with a good prognosis while enabling counseling, intensive systemic surveillance, and systemic adjuvant therapy to be targeted at high-risk patients.

Prognostic factors include anatomic, histologic, and genetic predictors.^2^ Anatomic predictors include largest basal tumor diameter (LBTD); tumor thickness; extraocular spread, especially if extensive; and ciliary body involvement.^3^^–^^5^ Histologic predictors include the presence of epithelioid cells, closed loops, and some other types of extravascular matrix patterns; high mitotic count; high microvascular density; and high number of tumor-infiltrating macrophages.^6^^–^^10^ Genetic predictors include a growing number of factors, such as chromosome 3 loss, chromosome 8q gain, *BAP1* aberration, a class 2 gene expression profile, *PRAME* abnormality, and *SF3B1* mutation.^2^^,^^11^^–^^17^

As with other cancers, the Tumor, Node, Metastasis (TNM) staging system of the American Joint Committee on Cancer (AJCC) has become the standard method for estimating the risk of disease-related mortality according to the stage of choroidal melanoma.^18^ The 8th edition of the staging system for choroidal and ciliary-body melanomas was developed with data from more than 7000 patients provided by members of the European Ophthalmic Oncology Group. This system stages tumors according to four anatomic predictors: basal tumor diameter, tumor thickness, ciliary body involvement, and extraocular spread.^19^^,^^20^ It was developed by empirically dividing basal tumor diameter and thickness into 3-mm × 3-mm fractions and grouping these into four size categories so that all fractions within a particular category showed similar survival probabilities (Kaplan–Meier estimator) and so that all of the size categories had similar numbers of patients. These groups were then subcategorized according to the presence of ciliary body involvement and extraocular spread. Subcategories with similar survival probabilities were iteratively combined to form six prognostic stages. The TNM staging system has three main limitations. First, it does not account for genetic predictors of metastasis, such as chromosome 3 loss, *BAP1* aberration, and class 2 gene expression profile. Second, it requires the practitioner to follow a number of steps to determine the stage of disease. Third, it does not account for competing risks, so that estimates of metastatic mortality are exaggerated due to use of the Kaplan–Meier estimator. This latter aspect is particularly relevant, because in frail populations, such as elderly subjects, other causes of death may occur prior to the occurrence of metastatic death.^21^

In 2007, we showed that prognostication is improved by combining multiple predictive factors.^22^ This insight led us to develop the Liverpool Uveal Melanoma Prognosticator Online (LUMPO), which estimates the absolute risk (i.e., cumulative incidence) of metastatic and non-metastatic mortality according to anatomic, histologic, and genetic data, also taking into account the patient's age and sex.^21^ LUMPO automatically computes the TNM tumor size category and stage of disease. A prototype of this tool is available online (www.LUMPO.net). It is easy and quick to use and has been validated internally and externally. A multicenter validation study has been completed.^23^

Because in some situations LUMPO is not available, we performed this study to develop a parsimonious, paper-based system that is easier and more convenient to use than the current TNM method.^18^^,^^24^ Our aims were also to take into account any available genetic data and to avoid bias caused by competing risks of death from other causes.

## Methods

### Inclusion and Exclusion Criteria

Patients were included in this study if they had been diagnosed with choroidal melanoma (with or without ciliary body involvement), confirmed histologically, and if they had been treated by enucleation, endoresection, exoresection, plaque radiotherapy, or proton beam radiotherapy at the Tennent Institute of Ophthalmology in Glasgow before 1993 or at the Ocular Oncology Service, Royal Liverpool University Hospital, between January 1993 and August 2018. They were excluded if they did not reside in England, Scotland, or Wales, because the National Health Service cancer registries provide the date and cause of death only for patients living in these parts of the United Kingdom.

### Investigations and Treatment

Preoperative investigation included full ocular and systemic history; slit-lamp examination and binocular indirect ophthalmoscopy; color photography; and B-scan ultrasonography, which was used to measure basal tumor dimensions and tumor thickness and to detect any extraocular tumor extension. Treatment was selected according to tumor size, location, and extent, as well as the patient's wishes and concerns, and consisted of various forms of radiotherapy, local resection, and laser therapy, individually or in combination, with enucleation reserved for patients whose tumor was considered too extensive for conservation of useful vision.^25^ Consent was obtained for the use of images, data, and tissues for research, teaching, and audit purposes.

### Laboratory Methods

Histopathological examination included light microscopy with hematoxylin and eosin staining, immunohistochemistry for melanoma with Melan-A, and, in tumors treated by enucleation, mitotic count per 40 high-power fields and evaluation of extravascular matrix patterns, such as closed connective tissue loops. Since 1998, we have offered patients genetic analysis of their tumors to detect lethal aberrations. Initially we used FISH^22^; however, in 2007, we replaced this method with multiplex ligation-dependent probe amplification (MLPA) and microsatellite analysis (MSA).^26^ These methods were more sensitive than FISH and required smaller samples, making it possible for us to perform prognostic biopsy of tumors treated with plaque or proton beam radiotherapy. Tumors were categorized as having (1) normal chromosome 3 and chromosome 8q; (2) chromosome 8q gain; (3) chromosome 3 loss; or (4) both chromosome 8q gain and chromosome 3 loss. Our criteria for considering these genetic aberrations to be significant were described previously.^22^^,^^26^ Patients found to have chromosome 3 abnormality were referred to a medical oncologist for systemic management, which involved liver imaging and long-term surveillance.^27^

### Postoperative Surveillance

After conservative therapy, we reviewed patients 6 months postoperatively, then every 6 to 12 months unless they lived far from our center, in which case they were discharged back to the referring hospital. Patients treated by enucleation received all follow-up at their local hospital unless they lived close to our hospital. Those with chromosome 3 loss were referred to an oncologist for systemic surveillance. Patients residing in England, Scotland, or Wales were flagged at the National Health Service cancer registries, which until September 2018 automatically notified us of the date and cause of death of all deceased patients. Cause of death was categorized as (1) definite metastatic uveal melanoma, (2) probable metastatic uveal melanoma, (3) possible metastatic uveal melanoma, (4) other malignancy, (5) non-cancerous disease, or (6) unknown. Fatality was coded as being caused by the uveal melanoma if metastatic disease from this tumor was considered to be “definite” or “probable” (i.e., when no other cause of death and no other source of metastases were specified).

### Statistical Methods

A backward-selection algorithm^24^ was applied to the original LUMPO model to identify the most informative predictors of the hazard rate of metastatic death. The relative importance of the risk factors was quantified using the rescaled Akaike Information Criterion (AIC),^24^ which estimates the relative quality of statistical models for a given dataset. This revealed that largest basal tumor diameter (LBTD) and chromosome 3 status (C3S) were the factors most predictive of metastatic death. We therefore considered two parsimonious models for evaluation and comparison with the TNM staging system: (1) LBTD alone for situations when genetic data are not available, and (2) LBTD combined with C3S. Using similar methods, we identified age as the most important predictor of death from other causes. Because we were interested in developing a simple estimator of absolute risks of death due to metastasis, we recoded LBTD into a six-level factor. C3S was expressed as a binary factor. Age at treatment was also coded into a binary factor by thresholding at 80 years, the age that maximizes the separation of the absolute risk of non-metastatic death from the absolute risk of metastatic death (Fig. b). When C3S was not known, C3S was estimated at the model fitting stage by applying an approximate Bayesian multiple imputation procedure as we did in the development of LUMPO.^21^

**Figure.**
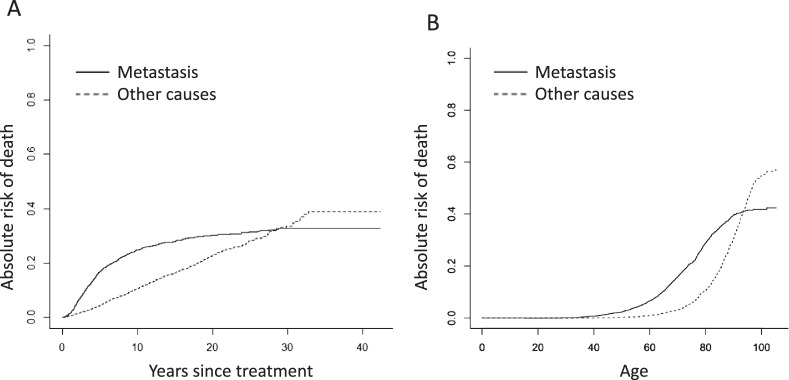
Absolute risk (cumulative incidence) of metastatic deaths and non-metastatic deaths according to (**a**) time after treatment and (**b**) age.

We defined the two models in terms of the Aalen estimator of absolute risk.^28^ This estimates risk of metastatic death while taking into account competing risks of death from other causes:

(1) $${\hat{F}}_{k}\left(t\right)=\int_{0}^{t}{\hat{S}}_{k}\left(u-\right)Y_{k}^{-1}\left(u\right)\;dN_{k}\left(u\right)$$

where *k* denotes the group (six groups for the LBTD-only model and 12 groups for the LBTD/C3S model), *N_k_*(*t*) is the number of metastatic death events by time *t*, *Y_k_*(*t*) is the number of subjects still at risk just prior to *t*, and ${\hat{S}}_{k}\left(t-\right)$ is the left-hand limit of the Kaplan–Meier estimate of all-cause survival. No assumption was made about the independence of the competing risk of death from other causes. In conformity with the LUMPO model, we used a Cox model for the competing risks of death due to other causes, with risk factor the binary age at treatment of the subjects.

Point estimates for the absolute risks at 2, 5, and 10 years from treatment for each group were obtained as the average of the absolute risks over the *M* = 101 imputed datasets:^29^

(2) $$\overset{-}{\underset{k}{\hat{F}}}\left(t\right)=\frac{1}{M}\sum_{i}{\hat{F}}_{k}^{\left(i\right)},\;M=101,\;t\in \left\{2,5,10\right\}$$

A 95% pointwise confidence interval (CI) for each estimated absolute risk was obtained by the pooled sample multiple imputation bootstrap technique to account for the uncertainty of the multiple imputation process.^29^ Bootstrap resampling was applied 2000 times for each imputed dataset. The 2.5 percentile and 97.5 percentile of the bootstrap estimates were used as the lower and upper confidence limits, respectively. The bootstrap procedure was also used to obtain estimates of discrimination accuracy, as expressed by the C-index (concordance index) for competing risks.^30^ This provides an assessment of the ability of the model to rank event times according to the individual risk scores. The C-index reflects the performance of the model, with values of 0.6, 0.7, 0.8, and 1.0 conventionally indicating poor, good, strong, and perfect predictive ability, respectively.

All of the analyses were performed using Microsoft R Open 3.5.3 (Redmond, WA, USA),^31^ with R programs written by one of the authors (AE) using the libraries *boot*, *pec*, *riskRegression*, *rms*, and *survival*. Parallel bootstrap computation of the multiple-imputation model required about 15 hours on an Intel Core i5 computer with 16 GB RAM.

Ethical approval for the study was obtained from the Health Research Authority South Central – Hampshire B Research Ethics Committee (REC Ref 15/SC/0611). We adhered to the tenets of the Declaration of Helsinki.

## Results

### Patient Demographics

The entire cohort was comprised of 8348 patients with choroidal melanoma; of these, 4174 had known chromosome 3 status, with monosomy 3 (M3) in 1705 patients and disomy 3 (D3) in 2469 (Table 1). The median LBTD was 12 mm (range, 2.4–28; interquartile range, 9.6–14.8). The median follow-up time was 7.1 years (range, 0.01–42.3; interquartile range, 3.2–13.6). A total of 1553 patients had died by the end of the study, with death attributed to metastatic disease in 956 patients, 816 of whom had M3 (Table 1). The Figure shows the absolute risks of metastatic and non-metastatic death according to time after treatment and age.

**Table 1.** Patient Chromosome 3 Status and Age at Treatment

	Chromosome 3 Status						Age at Treatment			
	Unknown		Disomy 3		Monosomy 3		<81 yr		>80 yr	
		Metastatic		Metastatic		Metastatic		Non-Metastatic		Non-Metastatic
LBTD (mm)	N	Deaths, n (%)	N	Deaths, n (%)	N	Deaths, n (%)	N	Deaths, n (%)	N	Deaths, n (%)
<10.1	1067	69 (6.5)	144	0 (0)	41	11 (26.8)	673	46 (6.8)	579	141 (24.4)
10.1–12.0	721	96 (13.3)	128	4 (3.1)	52	11 (21.2)	433	28 (6.5)	468	111 (23.7)
12.1–14.0	591	160 (27.1)	98	3 (3.1)	80	21 (26.3)	410	37 (9.0)	359	7 (20.9)
14.1–16.0	405	173 (42.7)	71	4 (5.6)	94	36 (38.3)	264	14 (5.3)	306	69 (22.5)
16.1–18.0	227	121 (53.3)	48	7 (14.6)	85	49 (57.6)	147	7 (4.8)	213	39 (18.3)
18.1–28.0	219	136 (62.1)	22	3 (13.6)	81	52 (64.2)	122	3 (2.5)	200	27 (13.5)
Total	3230		511		433		2049		2125	

The absolute risk of metastatic death accelerated during the first 5 postoperative years, reaching 25% at 10 years and settling at 30%, whereas the risk of non-metastatic death increased at a constant rate, ultimately reaching a level of 40%. Metastatic and non-metastatic deaths accounted for approximately 40% and 60% of all observed mortality, respectively, with the cumulative incidence of metastatic death accelerating almost 20 years earlier than that of non-metastatic death. The increase in non-metastatic mortality was especially marked after the age of 80 years.

### Predictors Most Informative of Metastatic Death

Table 2 shows the risk factors for metastatic and nonmetastatic death, ranked in order of decreasing importance, according to the AIC. LBTD and C3S were the most informative factors, with rescaled AIC values of 124 and 519, respectively. Sex, ciliary body involvement, and chromosome 8q status were not sufficiently informative to justify the increased complexity of the models, which would have occurred had they been included (negative AIC values denote an increased chance of model overfitting). The Cox model for competing risk of non-metastatic death depended only on age at treatment.

**Table 2.** Risk Factors for Metastatic and Non-Metastatic Death

Risk Factors	Akaike Information Criterion
Metastatic death	
Chromosome 3 status	519
Large basal tumor diameter	124
Closed loops	42.1
Extraocular spread	23.2
Epithelioid cytomorphology	16.0
Mitotic count	11.5
Age at treatment	8.02
Tumor thickness	3.25
Chromosome 8q status	–0.56
Ciliary body involvement	–1.73
Sex	–1.94
Non-metastatic death	
Age at treatment	657.93
Sex	4.43

### Mortality According to Basal Tumor Diameter and Chromosome 3 Status

Table 3 shows the absolute risks of metastatic death according to our two models, with 95% bootstrap CIs. The 10-year metastatic mortality ranged from 1.4% in older patients with a small D3 melanoma to 80% in younger patients with a large, M3 tumor, with intermediate values in patients with no genetic data. In general, the risk of metastatic death was higher in younger patients.

**Table 3.** Absolute Risk of Metastatic Death

	Absolute Risk (95% CI)					
	Age <81 yr at Treatment			Age >80 yr at Treatment		
	Years After Treatment			Years After Treatment		
LBTD, mm	2	5	10	2	5	10
	Disomy-3 Melanoma					
<10.1	0.1 (0, 0.5)	0.7 (0, 1.6)	2.1 (0.7, 4)	0.1 (0, 0.5)	0.6 (0, 1.4)	1.4 (0.4, 2.6)
10.1–12.0	0.8 (0.2, 1.7)	1.7 (0.5, 3.4)	3.7 (1.5, 6.7)	0.7 (0.1, 1.6)	1.5 (0.5, 3)	2.7 (1.1, 4.8)
12.1–14.0	1.1 (0, 2.5)	3.3 (1, 6)	6.5 (2.7, 11)	1 (0, 2.4)	2.9 (0.9, 5.3)	4.9 (2, 8.3)
14.1–16.0	1.5 (0, 4)	5.3 (1.8, 9.6)	11 (4.9, 18)	1.5 (0, 3.8)	4.6 (1.6, 8.4)	8 (3.7, 13)
16.1–18.0	3.1 (0, 8.7)	12 (5.1, 21)	17 (8, 28)	2.9 (0, 8.3)	11 (4.5, 19)	14 (6.5, 22)
18.1–28.0	6 (0, 14)	12 (2.4, 25)	18 (6.2, 32)	5.7 (0, 14)	11 (2.3, 22)	15 (4.6, 26)
	Monosomy-3 Melanoma					
<10.1	2.5 (0.6, 5.2)	13 (6.4, 21)	26 (13, 41)	2.4 (0.5, 5)	11 (5.5, 18)	19 (9.6, 30)
10.1–12.0	5 (2.2, 8.4)	20 (12, 28)	37 (22, 51)	4.8 (2.1, 8.1)	18 (11, 25)	28 (17, 38)
12.1–14.0	8 (4.9, 12)	33 (24, 42)	53 (38, 65)	7.9 (4.7, 12)	29 (21, 37)	42 (30, 51)
14.1–16.0	13 (9.2, 18)	42 (35, 50)	66 (55, 75)	13 (8.8, 17)	37 (30, 44)	52 (43, 60)
16.1–18.0	21 (16, 27)	59 (51, 67)	77 (67, 85)	20 (15, 26)	53 (46, 61)	64 (56, 71)
18.1–28.0	32 (26, 38)	70 (63, 78)	80 (73, 87)	31 (25, 37)	64 (57, 71)	71 (64, 77)
	Unknown Chromosome 3 Status					
<10.1	0.6 (0.2, 1.1)	3.1 (2.1, 4.2)	6.6 (5, 8.3)	0.6 (0.2, 1)	2.7 (1.8, 3.6)	4.7 (3.6, 6)
10.1–12.0	2 (1.2, 3)	7 (5.3, 8.9)	13 (10, 15)	1.9 (1.1, 2.9)	6.1 (4.6, 7.8)	9.7 (7.8, 12)
12.1–14.0	4 (2.6, 5.4)	15 (13, 18)	25 (21, 28)	3.8 (2.5, 5.1)	14 (11, 16)	19 (17, 22)
14.1–16.0	7.9 (5.7, 10)	25 (22, 30)	41 (36, 45)	7.6 (5.5, 9.8)	22 (19, 26)	32 (28, 36)
16.1–18.0	15 (11, 19)	43 (37, 48)	55 (50, 61)	14 (11, 18)	38 (33, 43)	46 (41, 51)
18.1–28.0	25 (21, 30)	56 (50, 62)	65 (59, 70)	24 (20, 29)	51 (45, 56)	56 (51, 62)

### Accuracy of Predictive Models and TNM Staging System

Metastatic death was predicted more accurately when C3S was known than when such genetic data did not exist, with metastatic death-specific C-indices at 2, 5, and 10 years of 0.85, 0.85, and 0.84 and 0.79, 0.77, and 0.74, respectively. The C-indices of the TNM staging system at 2, 5, and 10 years were 0.81, 0.79, and 0.76, indicating that it was slightly better than the model with LBTD alone but not as accurate as the model that also included C3S (Table 4).

**Table 4.** C-Index Indicating Discrimination Accuracy of Estimates of Metastatic Risk

	Years After Treatment		
	2	5	10
LBTD and C3S, C-index (95% CI)			
Known	0.85 (0.82, 0.88)	0.85 (0.82, 0.87)	0.84 (0.80, 0.86)
Unknown	0.79 (0.76, 0.82)	0.77 (0.75, 0.78)	0.74 (0.73, 0.76)
TNM, C-index (95% CI)	0.81 (0.78, 0.85)	0.79 (0.77, 0.81)	0.76 (0.75, 0.79)

## Discussion

### Main Findings

We have developed two nonparametric models estimating the risk of metastatic death in patients with choroidal melanoma, basing these on LBTD analyzed both with and without known C3S, also taking into account competing causes of death and patient age category. Compared to the TNM staging system, our predictive models had similar accuracy when C3S was unknown and were superior when C3S was known. We prepared a series of tables to make prognostication quicker and easier in busy clinical environments.

### Strengths and Weaknesses

The main strengths of this study are the large number of patients, the long follow-up, and the prospective data collection. The main weakness is that the certified cause of death was ambiguous for some death certificates and may have been false in some patients, especially in those who had a second primary malignancy. Other studies that include mortality data suffer from this problem, as it is impractical to perform postmortem examinations on all patients. Another weakness is that when we staged tumors according to the TNM system we did not subclassify them according to whether any extraocular tumor extension was greater or less than 5 mm in diameter; however, in view of the rarity of extraocular spread this did not detract significantly from our study.

### Discussion of Methods

Patients with unknown C3S comprised 77% of our cohort. These patients were nevertheless included in our study because many patients with choroidal melanoma are still being treated without genetic typing of their tumor because of small tumor size or because such investigation is not possible at their hospital. Furthermore, exclusion of such patients would have reduced our sample size and biased the analyses, because unknown C3S was not evenly distributed among the competing risks and censored events. For example, genetic typing was more likely to be omitted in patients with a small tumor; these patients had a lower risk of metastatic death, because the genetic tumor type was more likely to be D3. For the same reasons, we also included genetic data from FISH analysis even though this method was relatively insensitive as compared to MLPA, which became our preferred method in the latter part of this study.

We did not include histologic predictors in this study, because melanoma cytomorphology showed only borderline associations with metastatic death in our cohort and because categorization according to melanoma cell type is subjective and therefore likely to show variation between pathologists. We excluded mitotic count and extravascular matrix, because these cannot be assessed in the tiny biopsy samples on which prognostication is based in patients undergoing radiotherapy. The backward-selection algorithm and AIC scores provide statistical support for the decision to remove these predictors.

The prognostic significance of genetic tests depends on their sensitivity in detecting lethal aberrations, and this sensitivity varies among methods. The findings of this study therefore pertain only to uveal melanomas analyzed by FISH, MLPA, or MSA. We assume that our results can be extrapolated to other methods, such as next-generation sequencing and gene expression profiling; however, our methods would have to be adapted for other genetic tests if the sensitivity and specificity of these tests in detecting lethal genetic alterations are different from the techniques we deployed in our study.

### Discussion of Results

We found that the prognostic model based on LBTD alone performed reasonably well across the time range of 2 to10 years (C-indices, 0.74–0.79) without the inclusion of ciliary body involvement, tumor thickness, and extraocular spread. Cai et al.^32^ reported similar findings. These predictors would have improved prognostication slightly, as with TNM staging (C-indices, 0.76–0.81). In a previous study, we found that the association between extraocular spread and metastasis was relatively weak in comparison with LBTD, epithelioid cell type, closed loops, and mitotic count.^5^

We and several others have shown that survival prognostication is enhanced by combining LBTD with genetic data.^22^^,^^33^^,^^34^ This is because a significant proportion of small choroidal melanomas show lethal genetic aberrations and, conversely, such aberrations are not present in many large tumors.^3^^,^^33^

Interestingly, in the present study, we did not find chromosome 8q status to be strongly associated with metastasis (Table 2). This is possibly because the MLPA assay does not provide information pertaining to the precise number of copies of 8q, only whether there has been gain or loss of material. Other studies have shown that the association between 8q gain and metastasis mortality increases with the number of copies of 8q, especially when this number exceeds 3.^35^^–^^37^ Despite this, we feel that the compromises we have made in developing parsimonious models are unlikely to be clinically significant in view of discrepancies arising from methodological variations between clinicians and centers (e.g., measurement of tumor dimensions, obtaining mortality data).

### Prognostic Form

We have developed a form with a selection of tables for estimating prognosis according to the data available (Supplementary Fig. S1).

### Clinical Implications

When developing the next edition of the TNM staging system, the AJCC may wish to consider our approach to combining anatomic and genetic predictors of metastasis. This committee may also investigate whether to retain or omit predictors that lose significance (i.e., ciliary body involvement and tumor thickness) when genetic data are entered into their models, as we have shown in this study and as others have shown previously.^32^ If our approach is validated externally and adopted by the AJCC committee, it may increase the uptake of TNM staging if generally found to be more convenient than the current system, especially as it allows anatomic predictors to be combined with genetic data. Some authors favor gene expression profiling, but this is not widely available outside the United States.

We emphasize that our prognostic system is not intended as a substitute for LUMPO, which overcomes many of the limitations of Kaplan–Meier analysis. LUMPO allows multivariable analysis of anatomic, histologic, and genetic data, without splitting continuous data into categories, while also adjusting for missing data and competing causes of death so that it is more accurate. Further, by interpolating data, LUMPO provides mortality estimates for small subgroups of patients. It is likely that LUMPO will continue to evolve as more data accumulate, allowing inclusion of a wider range of predictors (e.g., gene mutational status).

### Research Implications

There is a need for external validation of our prognostic models with long-term mortality data from cohorts of patients from other ocular oncology centers. It is also necessary to compare our system with other prognostic methods, including LUMPO

There is also scope for comparing the diverse genetic methods promoted by different groups to test the assumption that our prognostic scores are relevant with such techniques.

It would also be important to investigate the prognostic value of adding other metastasis predictors to statistical models, such as *BAP1*, *PRAME*, *SF3B1*, and *EIF1AX*. Although these factors are known to be associated with metastatic disease from uveal melanoma, it cannot be assumed that they improve prognostication in comparison with models using only LBTD and C3S or only LBTD and gene expression profiling.

## Conclusions

This study confirms that estimating the risk of metastatic death from uveal melanoma is enhanced by multivariable analysis combining anatomic and genetic predictors, as has already been achieved with LUMPO. For situations where using LUMPO is not possible, we propose estimating metastatic risk according to LBTD, if possible also considering genetic predictors of metastasis and taking into account competing causes of death. We have devised prognostic tables that may facilitate mortality estimation in routine clinical environments if validated by multicenter studies.

## Supplementary Material

Supplement 1
