# Supplementary material for: Parsimonious Models for Predicting Mortality from Choroidal Melanoma
Source: Invest Ophthalmol Vis Sci. 2020 Apr 25;61(4):35. doi: 10.1167/iovs.61.4.35 (PMC7401884; doi:10.1167/iovs.61.4.35)
Supplement: Supplement 1 [file iovs-61-4-35_s001.pdf]

Risk of metastatic death from choroidal melanoma

Name:________________ Surname:__________________ DOB:____/____/____ No:________________

Tumour diam: ____ mm. Tumour thickness: ____ mm. Ciliary body involved: Yes / No; Extraocular spread: Yes / No

Chromosome 3 loss: Yes / No; Chromosome 8q gain: Yes / No; Genetics: MLPA / MSA / NGS /Other: ___________

Metastatic mortality risk: 2 years: _____ 5 years: _____ 10 years: _____

DISOMY-3 MELANOMA							
Treatment age <81 yrs				Treatment age >80 yrs			
LBTD [mm]	Years after treatment			LBTD [mm]	Years after treatment		
	2	5	10		2	5	10
<10.1	0.1 [0, 0.5]	0.7 [0, 1.6]	2.1 [0.7, 4]	<10.1	0.1 [0, 0.5]	0.6 [0, 1.4]	1.4 [0.4, 2.6]
10.1-12.0	0.8 [0.2, 1.7]	1.7 [0.5, 3.4]	3.7 [1.5, 6.7]	10.1-12.0	0.7 [0.1, 1.6]	1.5 [0.5, 3]	2.7 [1.1, 4.8]
12.1-14.0	1.1 [0, 2.5]	3.3 [1, 6]	6.5 [2.7, 11]	12.1-14.0	1 [0, 2.4]	2.9 [0.9, 5.3]	4.9 [2, 8.3]
14.1-16.0	1.5 [0, 4]	5.3 [1.8, 9.6]	11 [4.9, 18]	14.1-16.0	1.5 [0, 3.8]	4.6 [1.6, 8.4]	8 [3.7, 13]
16.1-18.0	3.1 [0, 8.7]	12 [5.1, 21]	17 [8, 28]	16.1-18.0	2.9 [0, 8.3]	11 [4.5, 19]	14 [6.5, 22]
18.1-28.0	6 [0, 14]	12 [2.4, 25]	18 [6.2, 32]	18.1-28.0	5.7 [0, 14]	11 [2.3, 22]	15 [4.6, 26]

MONOSOMY-3 MELANOMA							
Treatment age <81 yrs				Treatment age >80 yrs			
LBTD [mm]	Years after treatment			LBTD [mm]	Years after treatment		
	2	5	10		2	5	10
<10.1	2.5 [0.6, 5.2]	13 [6.4, 21]	26 [13, 41]	<10.1	2.4 [0.5, 5]	11 [5.5, 18]	19 [9.6, 30]
10.1-12.0	5 [2.2, 8.4]	20 [12, 28]	37 [22, 51]	10.1-12.0	4.8 [2.1, 8.1]	18 [11, 25]	28 [17, 38]
12.1-14.0	8 [4.9, 12]	33 [24, 42]	53 [38, 65]	12.1-14.0	7.9 [4.7, 12]	29 [21, 37]	42 [30, 51]
14.1-16.0	13 [9.2, 18]	42 [35, 50]	66 [55, 75]	14.1-16.0	13 [8.8, 17]	37 [30, 44]	52 [43, 60]
16.1-18.0	21 [16, 27]	59 [51, 67]	77 [67, 85]	16.1-18.0	20 [15, 26]	53 [46, 61]	64 [56, 71]
18.1-28.0	32 [26, 38]	70 [63, 78]	80 [73, 87]	18.1-28.0	31 [25, 37]	64 [57, 71]	71 [64, 77]

UNKNOWN CHROMOSOME 3 STATUS							
Treatment age <81 yrs				Treatment age >80 yrs			
LBTD [mm]	Years after treatment			LBTD [mm]	Years after treatment		
	2	5	10		2	5	10
<10.1	0.6 [0.2, 1.1]	3.1 [2.1, 4.2]	6.6 [5, 8.3]	<10.1	0.6 [0.2, 1]	2.7 [1.8, 3.6]	4.7 [3.6, 6]
10.1-12.0	2 [1.2, 3]	7 [5.3, 8.9]	13 [10, 15]	10.1-12.0	1.9 [1.1, 2.9]	6.1 [4.6, 7.8]	9.7 [7.8, 12]
12.1-14.0	4 [2.6, 5.4]	15 [13, 18]	25 [21, 28]	12.1-14.0	3.8 [2.5, 5.1]	14 [11, 16]	19 [17, 22]
14.1-16.0	7.9 [5.7, 10]	25 [22, 30]	41 [36, 45]	14.1-16.0	7.6 [5.5, 9.8]	22 [19, 26]	32 [28, 36]
16.1-18.0	15 [11, 19]	43 [37, 48]	55 [50, 61]	16.1-18.0	14 [11, 18]	38 [33, 43]	46 [41, 51]
18.1-28.0	25 21, 30]	56 [50, 62]	65 [59, 70]	18.1-28.0	24 [20, 29]	51 [45, 56]	56 [51, 62]

Damato B, Eleuteri A, Hussain R, Kalirai H, Thornton S, Taktak A, Heimann H, & Coupland SE.
Parsimonious models for predicting mortality from choroidal melanoma.
Invest Ophthalmol Vis Sci. 2020;61(4):35. https://doi.org/10.1167/iovs.61.4.35

(November 2019)
